# XPS Investigation on Improving Hydrogen Sorption Kinetics of the KSiH_3_ System by Using Zr-Based Catalysts

**DOI:** 10.3390/ma15217454

**Published:** 2022-10-24

**Authors:** Anish Tiwari, Shivani Agarwal, Kriti Shrivastava, Takayuki Ichikawa, Ankur Jain, Rini Singh

**Affiliations:** 1 Center for Renewable Energy and Storage, Suresh Gyan Vihar University, Jaipur 302017, India; 2 Department of Physics, JECRC University, Jaipur 303905, India; 3 Graduate School of Advanced Science & Engineering, Hiroshima University, Higashi-Hiroshima 739-8527, Japan; 4 Natural Science Centre for Basic Research & Development, Hiroshima University, Higashi-Hiroshima 739-8530, Japan

**Keywords:** silanide, metal hydride, ball milling, activation energy, catalyst, XPS

## Abstract

The superior hydrogen storage properties makes the KSiH_3_ system a potential hydrogen storage material for practical applications. A theoretical capacity of 4.3 wt% bring this material to the front line of all the available hydrogen storage materials; however, the activation barrier of the reaction restricts the system to absorb and desorb hydrogen reversibly at elevated temperatures even if the thermodynamics suggest its room temperature operation. Several catalysts have already been tested to enhance its kinetic properties. In this work, the efforts were made to reduce the activation energy by using Zr-based catalysts to the KSi/KSiH_3_ system. The value of activation energy was found to be lowest (i.e., 87 kJ mol^−1)^ for the ZrH_2_-added KSiH_3_ system. The mechanism of this improvement was investigated by using X-ray photoelectron spectroscopy (XPS).

## 1. Introduction

There are many different natural resources on Earth that can be used to generate energy. Generally, conventional energy resources can either be produced once or can take thousands of years to replenish themselves. Commonly harvested energy resources falling in this category are the fossil fuels. Their popularity can be attributed to their easy availability and technological feasibility. But the overexploitation of such non-renewable resources is causing global warming and other environmental issues due to the excessive release of greenhouse gases and other toxic emissions into the environment. Due to the rising energy demand and cost of such traditional energy resources from the past few decades, scientists have been searching for new energy materials. Hydrogen has been proven to be a clean and popular alternative energy resource, as it creates only water after combustion along with a huge amount of energy [1]. As compared to the conventional fuels, hydrogen is a viable option as a secondary energy source due to its creation of almost three times the gravimetric energy [2]. Despite the fact that hydrogen has a number of appealing qualities, a number of problems must be fixed before hydrogen infrastructure can reap its many advantages. Storage facilities must be effective and secure if hydrogen is to be used as a fuel.

The major hydrogen storage technologies are liquified hydrogen, gaseous hydrogen, and hydrogen storage in solid state. Due to various operational difficulties associated with the practical utilization of gaseous and liquid hydrogen, hydrogen storage in solid materials is considered as the most suitable option for hydrogen storage. In solid-state hydrogen storage, hydrogen chemisorption on the material is always more advantageous over the hydrogen physisorption, as in the latter method, the hydrogen desorption for onboard applications is difficult to achieve at room temperature. Additionally, during chemisorption, the solid substance interacts with hydrogen chemically (high interaction energy of 100–200 kJ/mol) and the strong chemical bonds allow for the storage of hydrogen, which is then released under specific conditions as a result of a chemical reaction.

Chemisorption of hydrogen on metal hydride and complex hydrides is the most celebrated and commercially feasible technique of hydrogen storage in solid state [3,4]. The scientists around the globe have always been enthusiastic for the search of new materials with following capabilities: (1) good hydrogen capacity; (2) fast absorption/desorption rate; and (3) ambient operational conditions.

Light alkali metal hydrides (for example, magnesium and lithium hydrides) have impressive storage capacity, but they are thermodynamically very stable and therefore not suitable for charging and discharging at ambient conditions. Several research findings indicate the lowering of hydrogen desorption temperature by the addition of suitable additives like silicon. However, even after the addition of silicon, practical applicability of MgH_2_ and LiH is limited only at high temperature [5].

Among all the complex alkali metal silanides (MSiH_3_, where M = Li, Na, K, Rb, Cs), KSiH_3_ has been investigated as a promising material due to its matching properties with the ideal requirements [6]. The thermal annealing treatment of starting elements in the suitable ratio leads to the synthesis of KSi. The suitable thermodynamic parameters allow the room temperature operation of KSi with 4.3 wt% hydrogen storage capacity. However, reversibility at ambient conditions is difficult to attain due to a very high activation barrier. It can absorb hydrogen at about 100 °C, but the hydrogen desorption occurs at 200 °C. In the very first attempt, the absorption/desorption rate was significantly improved by the use of carbon; however, this system was turned into KH and K-Si compound, eventually leading to the reversibility loss [6]. This happened due to the excess heat generation during exothermic hydrogen absorption by KSi system. A number of metal and metallic alloys have been employed to achieve desired kinetics of hydrogen sorption but their high cost and lower abundancy limits their application on large scale [7]. Because d-block elements are abundant on earth, are affordable, and have high activity, they are a good alternative for enhancing the slow kinetics of hydrogen-storage materials.

Different transition metal-based catalysts and nanocomposites were studied to improve the sorption kinetics of KSi—KSiH_3_ transformation. Jain et al. [6] addressed the abovementioned issue of disproportionation by proper heat management and using one of the transition metal-based catalysts (i.e., mesoporous Nb_2_O_5_ [8]). The Nb_2_O_5_ was observed as the most promising catalyst among all the catalysts due to its effectiveness in reducing the activation energy down to 63 kJ mol^−1^ in contrast to 142 kJ mol^−1^ for as-prepared KSi. Similar studies showcasing the successful utilization of catalysts were performed by using nanometals (i.e., Ni, Co, Nb [9]), NbF_5_ [10], iron-based compounds (i.e., FeO, Fe, Fe_3_O_4_ and Fe_2_O_3_ [11]), vanadium-based catalysts [12], and Ti compounds [13] etc. As a result of this approach, kinetics was improved, activation energy was reduced, and activation cycles were eliminated.

Zirconium (Zr) and its derivatives represent another family of excellent catalysts, similar to other transition metals. Their high performance toward hydrogen dissociation and diffusion, zirconium, and its alloys have the tremendous potential to function as hydrogen-transfer centres. Zhang et al. [14] reported that MgH_2_ coupled with ZrCo nanosheets desorbed 6.3 wt% hydrogen at 300 °C in 5 min, whereas it took only 10 min to absorb 4.4 wt% at 120 °C under 3 MPa pressure. In another work, Zr-Ni alloy was used to prepare composite with MgH_2_ and the resulting system desorbed 5.9 wt% hydrogen with 4 min, which is significantly fast [15]. Moreover, these composites were able to absorb hydrogen completely at 250 °C within 1 min. Another improvement was observed by M. Sherif El-Eskandarany et al. [16] who used metallic glassy Zr_2_Ni to catalyze MgH_2_ and showed 5.8 wt% H_2_ desorption in 2.5 min at 250 °C. Chen et al. [17] used DFT calculations to demonstrate the decoration of the ZrH_2_ nanocatalyst on the MgH_2_ surface and suggested the fast kinetics as a result of lattice distortion between ZrH_2_ and MgH_2_/Mg phases.

In another report, the synergetic effect of Zr_0.4_Ti_0.6_Co nanosheets along with carbon nanotubes was investigated by Zhang et al. [18]. It resulted in the quick release of almost 90% hydrogen from MgH_2_ within 10 min at 300 °C. The effect of ZrMn_2_ nanoparticles was also investigated by the same group, which reported the hydrogen release at a lower temperature (i.e., 181.9 °C [19]).

Motivated from the above results, this work is designed to study Zr-based catalysts’ added KSi/KSiH_3_ system with an anticipated hope of improvement. The catalysed KSi samples were prepared by adding ZrCl_4_, ZrF_4_, ZrH_2_, and ZrO_2_ by ball milling. Several techniques including XRD, SEM, XPS, DSC, TG were utilized to study the improvement in the sorption properties of KSi system.

## 2. Materials and Methods

Potassium silanide was synthesized by the heat-treatment method described earlier [8]. The potassium metal along with silicon powder (silicin was taken in a slightly excessive amount) was placed in a SS chamber. The chamber was sealed and heated at 500 °C for 72 h. All the catalysts (i.e., ZrCl_4_, ZrF_4_, ZrH_2_, and ZrO_2_ (10 wt%)) were added to the prepared KSi by the mechanical milling method. For this, the as-prepared KSi sample along with the catalyst was transferred to milling vial and this vial with 10 Cr steel balls was sealed tightly inside the glove box. The Fritsch P7 instrument was used to perform milling at 400 rpm speed for a duration of 1 h. The X-ray diffraction (XRD) study was performed by using Rigaku (Tokyo, Japan), RINT-200, equipped with CuKα radiation. The XRD samples were prepared on a glass plate and were covered with a polyimide sheet (Dupont–Toray Co., Ltd., Kapton) inside the glove box. The energy dispersive X-ray (EDX) analysis combined with scanning electron microscopy (SEM) on JEOL, and a JSM6380A instrument was used to reveal the catalyst distribution and morphology. The protected samples (covered by a polyimide sheet inside glove box) on carbon tape were directly placed into an SEM chamber through a unique transferring device without any contact with air or moisture. As part of our evaluation of the KSi-KsiH_3_ system, the differential scanning calorimetry method (TA instruments Q10PDSC) and a combination of TG, DTA, and TDMS (Angelva, M-QA 200TS) methods was used for the study of hydrogen storage properties. The oxidation state of catalyst was evaluated by using X-ray photoelectron spectroscopy (XPS) by using a Thermo Fisher Scientific ESCALAB 250 Xi instrument (Walthamm, MA, USA) equipped with Al- Kα (1486.6 eV) X-ray source. The samples were loaded in a specially designed transfer chamber inside the glove box, and then this transfer chamber was connected to the XPS instrument. This arrangement ensures that the contact with atmospheric air and moisture by the samples is avoided.

## 3. Results

### 3.1. Structural and Morphological Characterization by XRD/SEM

**Figure 1:**
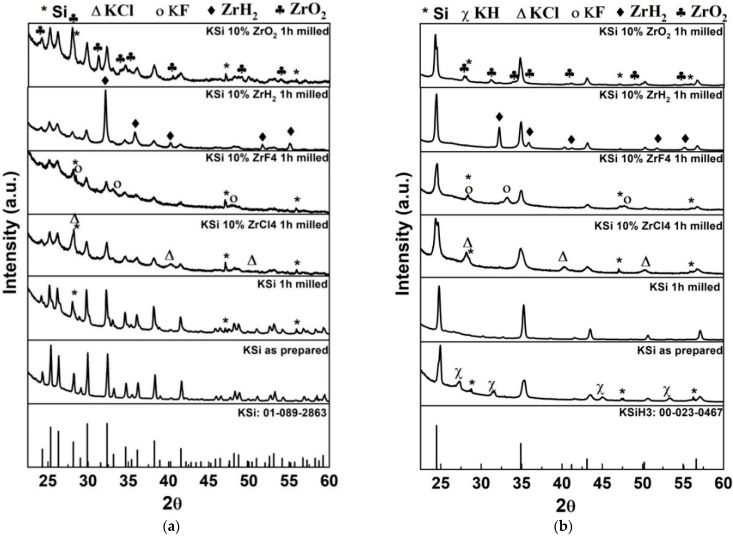
XRD pattern of (**a**) as prepared (**b**) hydrogenated KSi samples with and without catalysts. The phases are shown as Si (*), KCl (Δ), KF (o), ZrH_2_ (◆), ZrO_2_ (♣), and KH (χ).

To identify phases and characterize the structural properties of all the samples (pristine KSi milled with and without catalysts), XRD technique was used, and the results are shown in Figure 1. It is evident from the figure that no peaks are observed associated with potassium phases in any of the samples, which suggests that primary phases have been converted completely into the anticipated final phase of KSi. Minor Si peaks are observed, which is entirely normal, as Si was added during KSi preparation. After milling, the intensity of all peaks corresponding to KSi phase are decreased significantly, which was obvious due to the decrease in particle size after milling. A lowering and broadening of the peaks is observed for milled KSi with catalysts. A few additional peaks related to catalysts are also evident for all the samples except in the case of ZrCl_4_ and ZrF_4_. Instead of the catalyst peaks in these samples, KCl and KF peaks are visible, which originated as a result of the reaction between KSi and ZrCl_4_, ZrF_4_, respectively (i.e., disproportionation of KSi is occurred with these catalysts). This finding is quite similar to our previous results on VCl_3_ [12], TiCl_2_, TiCl_3_, and TiF_4_ [13]-added KSi samples wherein the appearance of KCl and KF phase was observed after milling.

The hydrogenation during DSC experiments (shown later in this manuscript) took place, and the samples were examined through the XRD experiment. The KSi phase-related XRD peaks have disappeared and KSiH_3_ peaks are grown up (Figure 1b), which confirmed the hydrogenation of these samples. The catalysts were remained intact during the hydrogenation as confirmed from the XRD peaks of these phases. The presence of KH is also evident for hydrogenated KSi sample in Figure 1b, which indicates the possibility of disproportionation during the harsh activation process. It is important to note that the excess amount of Si was added to avoid the disproportionation during the KSi preparation. The disproportionation during hydrogenation is totally different from that and caused by the excess heat which promoted the KH formation during the process of KSi to KSiH_3_ conversion. This excess heat could not be managed during the activation process of pristine KSi sample due to harsh activation procedure. When the KSi sample was milled with the catalysts, the activation process is not needed, and so the heat management could be carried out nicely, and the catalysed samples didn’t show any KH peaks.

**Figure 2:**
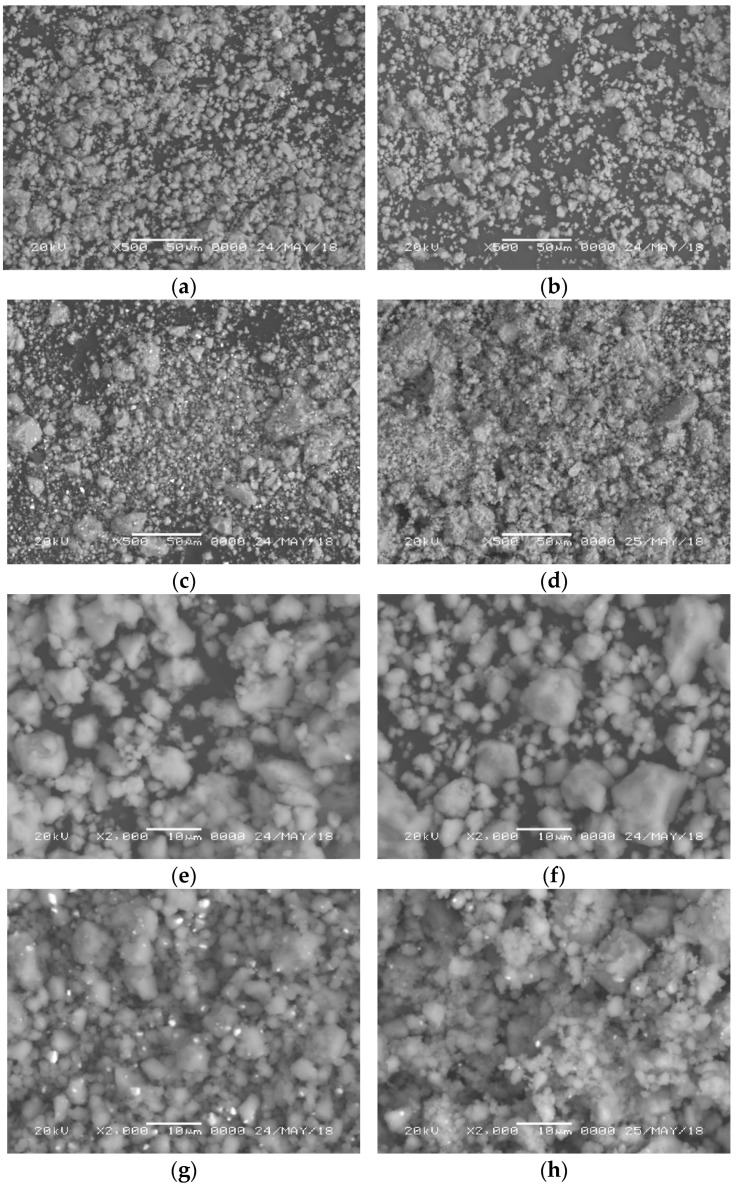
SEM images of (**a**) KSi–10%ZrCl_4_; (**b**) KSi–10%ZrF_4_; (**c**) KSi–10%ZrH_2_; (**d**) KSi–10%ZrO_2_ (secondary electron mode); (**e**) KSi–10%ZrCl_4_; (**f**) KSi–10%ZrF_4_; (**g**) KSi–10%ZrH_2_; and (**h**) KSi–10%ZrO_2_ (back-scattered electron mode).

In Figure 2a–d, the morphology of catalyzed KSi samples is shown. The morphology indicated the oxygen-free surface, which indicates that the experimental conditions were favorable. The particle size is observed to be almost similar, in the range of 1–50 µm for all the samples. Figure 2e–h shows the backscattered electron images of these samples at higher magnification. The reaction between KSi and ZrCl_4_, as well as ZrF_4_, is confirmed from the images, as is evident from no significant contrast (Figure 2e,f). On the other hand, white dots are present in Figure 2g,h, which correspond to Zr species as analyzed from EDX. It is clear that ZrH_2_ is distributed throughout the surface of KSi more homogeneously in comparison to that for ZrO_2_.

### 3.2. Hydrogen Sorption Studies by Differential Scanning Calorimetry (DSC) and Thermogravimetric (TG) Analysis

**Figure 3:**
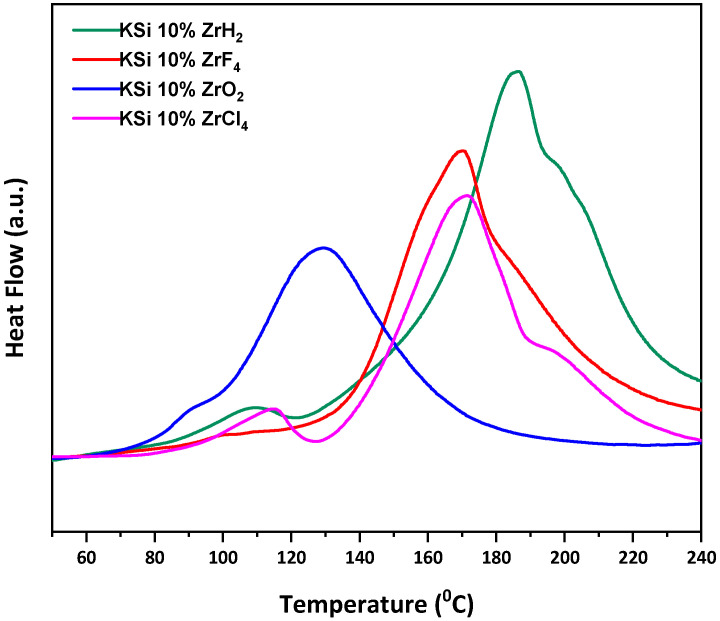
DSC profile of catalyzed KSi systems under 5 MPa H_2_ at heating rate 5 °C/min.

The DSC experiments with a scan rate of 5 °C/min were performed to study the hydrogen absorption properties of the samples under 5 MPa H_2_. Figure 3 describes the exothermic curves, which confirms the hydrogen absorption by the samples. The pristine KSi was observed as inactive in our previous reports [8,9,11,12,13], as there was no exothermic peak seen for pristine KSi. An exothermic peak at 105 °C was observed only after several activation cycles on KSi. In this work, the catalytic effect is clearly observed as the exothermic peaks were shifted to lower temperature for all Zr catalyst-added samples. The peaks were located in the temperature range 90–110 °C. By comparing all the Zr-based catalyst (i.e., ZrCl_4,_ ZrF_4,_ ZrH_2_ and ZrO_2_ in terms of exothermic peaks), ZrO_2_ was found best with the shoulder peak at lowest temperature that was 90 °C. The absorption behaviour on the basis of peak temperature (90–100 °C) is almost similar as that of other transition metal-based catalysts reported in our previous works [8,9,11,12,13]. On comparing all the catalysts studied so far, a slight change can be highlighted on the basis of onset hydrogenation temperature where it is observed that Fe_2_O_3_ is the best catalyst which reduced the onset temperature of hydrogenation down to 53 °C [11].

**Figure 4:**
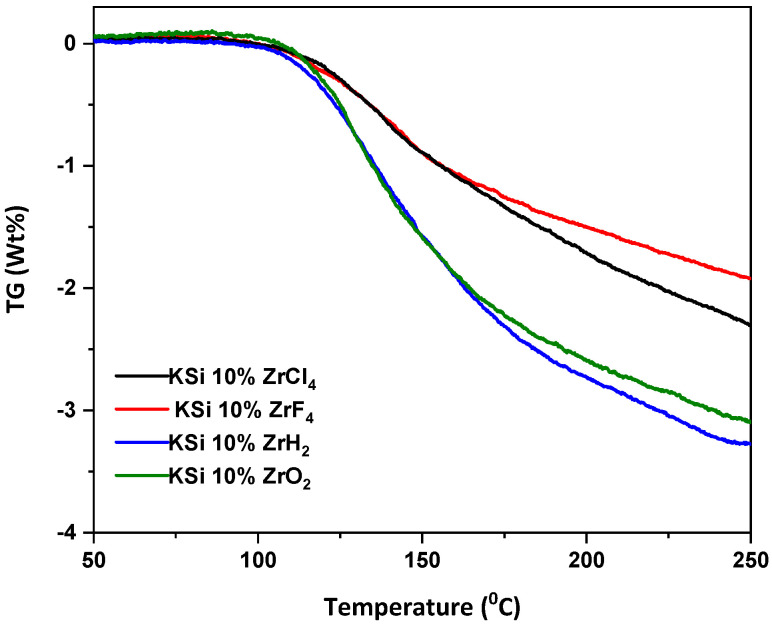
TG profile of catalysed KSi systems at heating rate 2 °C/min under 0.1 MPa Ar.

Following DSC measurements, thermogravimetric analysis (TG) was conducted to determine the total hydrogen content of all samples in a temperature range from RT to 250 °C with a heating rate of 2 °C/min and the results are depicted in Figure 4. The TG profiles clearly indicate the onset desorption temperature as 100 °C for ZrH_2_ and ZrO_2_-added samples with the total weight loss of 3.3 wt% and 3.2 wt%, respectively. The values are relatively close to those of the theoretical capacity of catalyzed KSi (3.9 wt%). The KSi sample added with ZrF_4_ could desorb only 1.8 wt% hydrogen. This is due to the formation of KF, which does not contribute toward hydrogen capacity; hence, the total effective hydrogen capacity reduces.

On reviewing the literature, it is observed that the desorption at higher temperature is caused either due to thermodynamic reasons or kinetic reasons. In this case, the thermodynamics of the KSi/KSiH_3_ system is favorable for its room temperature operation, so the high temperature operation observed in this work must be due to kinetic reasons. The kinetics of sorption can be understood in terms of activation energy. Thus, the activation energy of these catalyzed samples was evaluated by using Kissinger’s equation:

$$\ln K=-\frac{E_{\mathrm{des}}}{{\mathrm{RT}}_{p}}+ln\frac{{\mathrm{Rk}}_{0}}{E_{\mathrm{des}}}$$

where, *K* = *β/T_p_*^2^, *β* is heating rate, *T_p_* is peak temperature, *R* is gas constant, and *E_des_* is activation energy.

**Figure 5:**
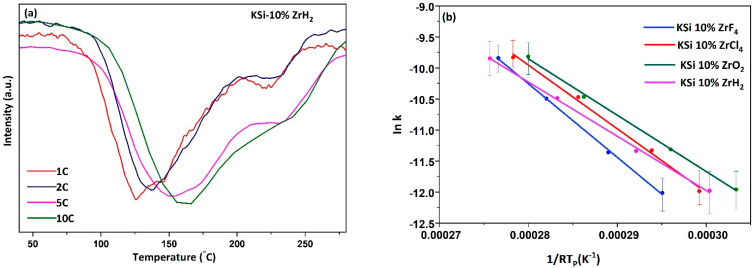
(**a**) DTA signals of KSi-10% ZrH_2_ at different heating rates. (**b**) Kissinger plots for KSi samples with catalysts.

To draw the Kissinger curves, the catalyzed KSi samples were allowed to desorb hydrogen at different scanning rates. The DTA signal corresponding to each heating rate was utilized to plot these curves. Figure 5a depicts the DTA curves of KSiH_3_-10% ZrH_2_ sample, whereas the corresponding Kissinger curves for the catalyzed KSiH_3_ samples are sketched in Figure 5b. The calculated activation energies for all the studied samples are summarized as follows: 102.7, 118.6, 87.4, and 90.8 kJ mol^−1^ H_2_ for ZrCl_4,_ ZrF_4_, ZrH_2_, and ZrO_2_ added KSiH_3_, respectively, as compared with the measured value, which was 142 kJ mol^−1^ H_2_ for the pristine KSi sample (Table 1). On comparing all the values, the ZrH_2_-added KSiH_3_ sample exhibited the lowest value of activation energy. It makes ZrH_2_ as the leading contender to improve the kinetics of KSiH_3_ system with ZrO_2_ as a runner up. Although by the use of Zr-based catalysts with the KSi/KSiH_3_ system, the activation energy is significantly altered, the mesoporous Nb_2_O_5_ remains the most effective and superior catalyst compared with other catalysts that have been studied in the KSi/KSiH_3_ system with the lowest activation energy of 63 kJ mol^−1^ [11].

### 3.3. XPS Investigation for Mechanism of Zr-Based Catalysts

**Figure 6:**
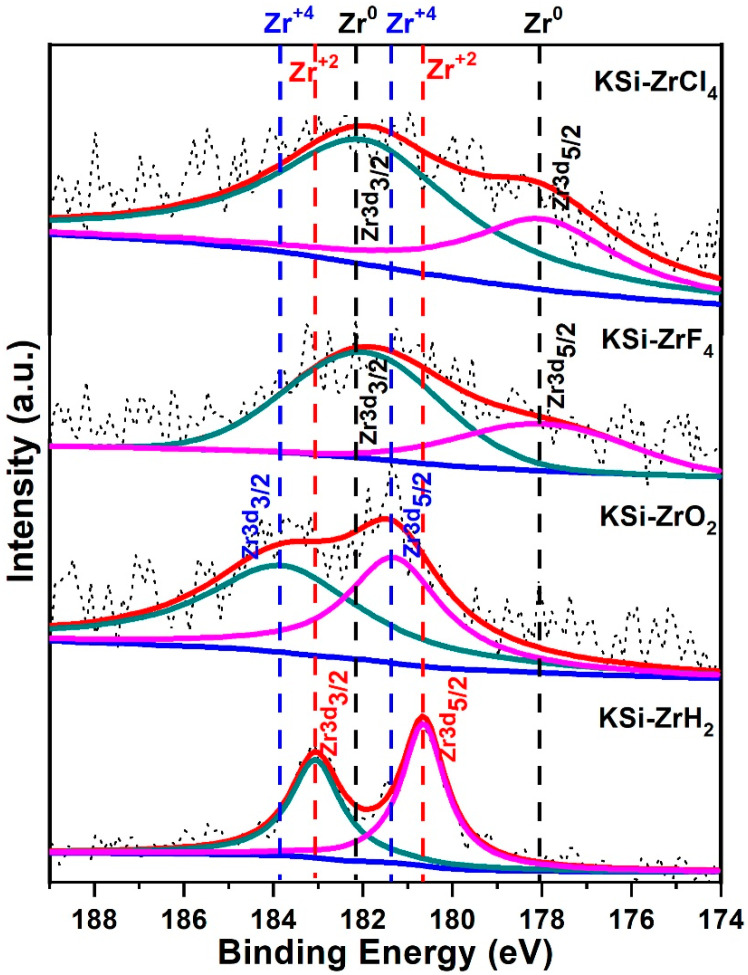
Core level Zr-3d XPS spectra for catalyzed KSi samples.

The catalytic mechanism of Zr-based catalysts was further explored by using X-ray photoelectron spectroscopy (XPS) and the core level Zr-3d spectrums for all the catalyzed samples are shown in Figure 6. All the samples showed characteristic doublet peaks. The ZrCl_4_ and ZrF_4_-added sample showed the peaks at 182.1 and 178.1 eV corresponding to 3d*3/2* and 3d*5/2*, respectively, which corresponds to metallic Zr state (Zr^0^). This is in line with the XRD observations, where the formation of KCl and KF were confirmed after milling, but no peaks corresponding to Zr were visible. XPS results confirmed the presence of Zr and so the speculated reaction is confirmed. Although the formation of the metallic state, in case of other metals, has been suggested as being helpful in enhancing the kinetics of hydrogen sorption of many hydrogen storage materials [12], the performance here is not promising. The activation energies for both the samples (ZrCl_4_ and ZrF_4_) were found at higher values. This might be due to the presence of KCl and KF which probably acted as poison for the surface of KSi samples and prevent the easy hydrogenation of these samples. On the other hand, the KSi sample milled with ZrO_2_ exhibited doublet peaks at 183.9 and 181.4 eV suggested the presence of Zr^+4^ state, which is obvious. Lastly, the ZrH_2_-added KSi sample showed the doublet peaks at 183.0 and 180.6 eV, which corresponds to the Zr^+2^ state and are characteristic peaks of ZrH_2_ phase. The KSi sample milled with ZrH_2_ delivered the best performance with the lowest value of activation energy i.e., 87 kJ mol^−1^. All the above analysis brings us to a conclusion that the Zr species with oxidation state of +2 is superior among all the studied samples. It is noteworthy that in our previous report on V-based catalyst [12], the in situ-formed metallic state of vanadium from vanadium oxide was found to have the best catalytic effect. However, in the present work, metallic zirconium is observed only in the case of ZrCl_4_ and ZrF_4_, whereas ZrH_2_ or ZrO_2_ could not be reduced to metallic Zr. It is imperative to note that the oxidation state 0 (metallic Zr), generated in the ZrCl_4_ and ZrF_4_-added samples could also be the leading catalyst; however, the possible poisonous effect of KCl and KF pulled these catalysts (ZrCl_4_ & ZrF_4_) back in the race.

## 4. Conclusions

Potassium silanide is a complex alkali metal hydride and a potential hydrogen storage material due to its theoretical storage capacity of 4.3 wt% and optimum thermodynamics, but a major disadvantage associated with it is the high activation energy of hydrogen sorption processes. The addition of the catalyst to these metal hydrides is one of the effective ways to improve the hydrogen sorption kinetics. The variable valences of d-block elements is a specific feature. At specific temperatures and pressures, induced vacancies may assist in absorbing hydrogen in empty space and releasing it as necessary. Their elemental as well as compound form, transition metals, are effective catalysts.

In the present work, requirement to perform activation cycles before hydrogen storage is eliminated by the addition of a Zr-based catalyst. This catalyst effectively reduced the activation energy and enhanced the hydrogen sorption kinetics. The use of the ball milling technique for the mixing of additive further enhanced the sorption rate. Among all the Zr-based catalysts ZrH_2_ followed by ZrO_2_ were found to be the leading materials which reduced the activation energy to 87 and 91 kJ mol^−1^ in comparison to 130 kJ mol^−1^ H_2_ for the KSi sample without the catalyst. The XPS results confirmed the superiority of Zr^+2^ species as the best catalyst among all the studied samples. The result is slightly different from our earlier results on a vanadium-based catalyst, in which the metallic state of the catalyst was found to be responsible for the enhancement. Here ZrCl_4_ and ZrF_4_ catalysts reduced to a metallic state; however, the performance is not as expected, probably due to the formation of inactive KCl and KF phases.

## Figures and Tables

**Table 1:** Activation energies of Zr-based catalysts (with milled samples).

Sample	Activation Energy (KJ mol^−1^)
KSi-ZrCl_4_	102.7
KSi-ZrF_4_	118.6
KSi-ZrO_2_	90.8
KSi-ZrH_2_	87.4

## Data Availability

Data available with the corresponding author.
